# The Flipped Learning and Blendspace to Improve Pupils’ Speaking Skills

**DOI:** 10.3389/fpsyg.2022.866270

**Published:** 2022-08-03

**Authors:** Cassandra Santhanasamy, Melor Md Yunus

**Affiliations:** ^1^Faculty of Education, Universiti Kebangsaan Malaysia, Bangi, Malaysia; ^2^SK Sementa Klang, Klang, Malaysia

**Keywords:** flipped learning, Blendspace, speaking skills, education, effective pedagogy

## Abstract

During the COVID-19 pandemic, the continuity of teaching and learning is very important to provide sustainable education to all pupils. The most difficult aspect of language acquisition has always been the speaking component. Pupils’ lack of interest and the difficulty in teaching and practicing speaking skills in the traditional classroom are the main issues that hinder pupils’ speaking skills. Thus, the purpose of this study was to explore the flipped learning approach to improve primary school pupils’ speaking skills. In this study, Blendspace was used to support the flipped learning method to help pupils improve their speaking skills. This study employed a mixed-method research design. For pre–posttests, questionnaires and interviews were used to collect data from Year 3 primary school students. The findings revealed that the use of Blendspace in teaching speaking has improved pupils’ speaking skills and that the pupils were motivated to practice speaking inside and outside of the classroom. Hence, the utilization of Blendspace in the flipped learning approach is an effective pedagogy to improve pupils’ speaking skills. The findings of this research would be useful for teachers and policymakers to assist pupils in enhancing their speaking abilities.

## Introduction

The COVID-19 pandemic has challenged educational institutions and policymakers to ensure the continuity of education. Educators need to adapt to the transitional shift in the education system. The English language has become significantly important and has greatly contributed to many educational sectors. As language is essential in today’s interconnected world, it is vital to be linguistically competent and possess communicative language abilities. As language learning is a complex process, speaking is the most important skill that English as Second Language (ESL) pupils need to master.

In Malaysia, the Ministry of Education (MOE) has implemented the Malaysian Education Blueprint (2013–2025) that aims to transform the education system (Ministry of Education, 2015). One of the aims of this blueprint is to ensure that pupils are proficient in both the Malay language and the English language. Nevertheless, some initiatives were taken to foster pupils’ communication skills so that they could be competent speakers in the future. The Common European Framework of Reference for Languages (CEFR) was created to standardize language proficiency across several countries. The CEFR introduced aims to ensure that pupils could communicate confidently, proficiently, and competently. The Common European Framework Reference (CEFR) was created to provide students with the language they will use in their everyday lives (Samat et al., 2019).

The Malaysian Ministry of Education aims to achieve the nation’s aspirations to produce young competitive generations. The school-based assessment (SBA) required teachers to assess pupils’ speaking skills with a score of 1–6, which ranges from low-proficiency level to the advanced level. In Malaysia, the SBA was introduced to replace the centralized traditional examinations. It requires the active involvement of teachers to assess their pupils’ language skills in the English language. Speaking assessment plays a majority role in evaluating and motivating pupils. A speaking assessment rubric could assess pupils’ fluency, pronunciation, vocabulary, and grammar. A study by Timpe-Laughlin et al. (2020) reported that Spoken Dialogue Systems (SDSs) were used to engage pupils with speaking tasks. The teachers agreed that SDSs promote a fun and interactive environment for L2 speaking practice. Fan and Yan (2020) suggested that diagnostic speaking assessment should be increased to provide better feedback to the pupils so that they could improve their speaking skills. Hence, the speaking assessment plays an equal role as the CEFR assessment. In this globalized world, education is no longer restricted to a physical classroom setting (Rafiq et al., 2020). Incorporating technology into the flipped classroom could increase pupils’ autonomy to learn better to improve their speaking skills. As the flipped classroom develops autonomous learners and an adaptable learning environment in which students can perceive knowledge, it is best suited for a language class (Santhanasamy and Yunus, 2022).

In this research, the constructivism theory addresses the student-centered learning theory whereby pupils are regarded as the active agent in participating and interacting with their peers. This shift in the roles of pupils and the teachers promotes active learning in language skills. The teacher acts as the guide and facilitator in assisting the pupils to participate in the flipped learning approach. Therefore, this framework is useful in conceptualizing the flipped learning approach into a more independent and autonomous role in the learning process. Grounded on the social constructivism theory, the flipped learning approach promotes an interactive, student-centered learning environment for the pupils that could improve pupils’ speaking skills.

Social constructivism theory was founded by Lev Vygotsky. This foundation of this theory is based on Vygotsky’s Zone of Proximal Development (ZPD). Whenever a student is in the zone of the proximal development area of any task, the appropriate support should be given to motivate them to accomplish the task (Vygotsky, 1978). As students share knowledge, ideas, and opinions, they began to accept and analyze the information socially. Joseph and Joy (2019) identified that learning is an active process whereby pupils construct their knowledge and relate them to their prior experiences and knowledge from a social constructivist point of view. This social construction of knowledge through the FA approach enables collaboration between one another to achieve the speaking goals.

In addition, Lev Vygotsky discovered a mutual relationship between social activities and cognitive processes that led to the sociocultural theory of development. Pupils participate and solve problem-based questions beyond their topic with the support of their teachers and peers (Vygotsky, 1978). Flipped learning incorporates a social constructivist learning approach as collaborative group activities to learn the concept. Pupils can work in teams and share multiple perspectives in the learner-centered environment. Thus, pupils became active constructors in the learning process particularly when they are immersed in the FL approach.

Furthermore, providing scaffolding to pupils enables them to bridge the understanding of the known and unknown learning content. This theory promotes engagement to optimize pupils’ learning outcomes as well as to practice speaking with their peers and teacher. The teacher acts as the facilitator and guides pupils in the activities. Appropriate learning activities are given based on the pupil’s level of difficulty and learning styles, thus encouraging peer interaction. Grounded on the social constructivism theory, the FL approach promotes active learning and a student-centered learning environment among the pupils in language learning. There are two theoretical frameworks based on these studies: Bloom’s Taxonomy (Santosa, 2017; Abedi et al., 2019; Chen and Hwang, 2020; Sargent and Casey, 2020) and constructivism theory (Singh et al., 2018; Erdemir and Yangın-Ekşi, 2019; Yeşilçınar, 2019). They help to facilitate and monitor the speaking performance of the pupils.

## Literature Review

### Speaking Skills

The capacity to communicate effectively has long been held up as a barometer of a successful individual. Other linguists and academics have various perspectives on speaking abilities. According to Brown (2007), speaking is an active process that produces and receives sounds to construct meaning. Thornbury (2005) states that speaking is the ability to communicate with others in daily life. We need to be active listeners and respond to the communication. Hence, responding to a conversation implies that the listener understood the speaker’s intention or message. Learners can express themselves, share their thoughts and feelings, and comprehend and inquire in this way (John and Yunus, 2021). The capability of pupils to converse in English with proper pronunciation, grammar, vocabulary, and fluency is used to determine their progress in speaking English (Tiing and Yunus, 2021). In speaking, there are a few important components that should be known. According to Harris (1974), there are five components of speaking skills such as pronunciation, grammar, vocabulary, comprehension, and fluency. Thus, these components provide a better understanding and transition of the communication process.

In the second language acquisition theory, Krashen (1985) expressed that speaking skills are the output of a language and it takes a longer time to develop this skill. Therefore, among the four language skills, speaking skills were considered the most difficult skill in language learning, especially among the L2 learners. Speaking skills have always been a challenging task for the L2 learners. To interact, more importantly, L2 learners need more mastery of the language, such as grammar, vocabulary, and phonology to increase their confidence to speak more. The importance of speaking skills enables pupils to converse well, as well as to face the twenty-first century challenges. Similarly, Nunan (2003) highlighted the speaking skill as a productive skill, and it produces systematic verbal utterances to convey meaning. Pupils can easily share knowledge and information if they possess good speaking skills (Mahendra et al., 2020). Moreover, communication is one of the four elements of 4Cs in the twenty-first century skills (Paneerselvam and Mohamad, 2019). This ensures pupils compete globally and build their self-confidence level. Brown (2004) states that assessment is a continuous performance in measuring pupils’ speaking level. Hence, teachers should be knowledgeable in evaluating the speaking proficiency of pupils. They are responsible for conducting various means of evaluating speaking for better engagement.

In Malaysia, English is regarded as the second most important language after the Malay language. Most of the English as Second Language (ESL) pupils are struggling to develop their speaking skills. There are two issues that lead to the need to implement the flipped learning approach. There is a lack of interest and knowledge in improving speaking skills, and also, there is difficulty in teaching and practicing speaking skills in the traditional classroom. Firstly, the pupils’ lack of interest to practice the language had caused poor speaking performances in language learning. They are not confident to express their opinions and ideas. Lack of vocabulary knowledge and pronunciation mistakes lowered their self-esteem to use the English language to interact with their peers and teachers. Moreover, the students have had little exposure to the English language due to their family origin and culture. As their family members do not communicate in English, the pupils have low opportunities to speak and neglect the language. During this COVID-19 pandemic, it is important to prolong the continuity of the teaching and learning process, especially in their speaking skills. Pupils should not be abandoned and would lose their motivation to speak due to limited exposure and interest. This shows that there is a need to guide the pupils to use the language and improve their speaking skills through the flipped learning approach.

### Flipped Learning

In addition, the flipped learning approach is a combination of virtual and face-to-face teaching methodologies that combine inside and outside class interaction. This approach is gaining more attention among educators from different fields and levels around the world. The flipped learning method includes two phases: the pre-class learning and in-class learning phases (Khasanah and Anggoro, 2022). Despite the increased interest, teachers’ lack of technological knowledge remains a constant barrier to the flipped learning approach in teaching multiple language areas (Santhanasamy and Yunus, 2022). Similarly, students’ ability to be more responsible for their own learning behavior has risen as a result of the transition effect of the traditional classroom to a student-centered approach. Hence, a fun and interactive learning environment ensures pupils to be active and engage in the speaking lesson.

Educators must change their teaching and learning strategies to a more flexible and inventive methodology that will allow language learning and abilities to develop consistently. Quality teaching resources are created through the use of an online platform, and teaching methods have evolved to include a wide range of methods and strategies, as well as the creation of teaching during times of crisis. Teachers’ lectures from traditional classrooms are converted into video clips for students to see before class (Lin, 2021). As a result, the flipped learning method offers both synchronous and asynchronous learning options. The Flipped Learning Network (2014) has described the four pillars of the flipped learning approach as an acronym, F-L-I-P. The four pillars are flexible environment, learning culture, intentional content, and professional educators. This suits the role of teachers and pupils to participate actively in the teaching and learning processes.

Furthermore, the foundation of the flipped learning approach focuses on student-centered learning. Flipped learning converts regular classroom lectures into video clips that students can see before class (Tiing and Yunus, 2021). In our globalized society, the flipped learning approach of education is flexible and is no longer restricted to classroom instruction. The flipped classroom gives students additional offline and online exposure to the language, which can help the pupils with speaking activities while also improving higher-order thinking skills (HOTS) (Riza and Setyarini, 2019). Bloom’s (1984) taxonomy includes six levels of cognitive processes: remember, understand, apply, analyze, evaluate, and create. It is arranged from the lowest level to the highest level. In the traditional classroom, teachers deliver lectures on the topic and pupils are involved in the lower cognitive levels of Bloom’s Taxonomy, namely remembering and understanding. In contrast, in the flipped classroom, the content delivery is delivered outside the classroom whereby pupils watch the video recording and access the learning materials at their own pace. The act of remembering and understanding could be practiced and repeated at the pupil’s own pace. In the classroom, teachers provide guidance and collaborate with their peers in performing the higher levels of Bloom’s Taxonomy for better engagement (Wei, 2019). As a result, Bloom’s Taxonomy assists the flipped learning strategy in ensuring the flipped classroom’s success in boosting HOTS.

Active learning refers to the process in which pupils engage in activities such as writing, discussion, or problem-based tasks that promote understanding and evaluation of classroom contents. Some researchers who examined the flipped learning concept with active learning (Parra-González et al., 2020; Sargent and Casey, 2020), agreed that active learning plays a fundamental aspect in facilitating the flipped learning approach. When students watch the lesson video at home and discuss the content in the classroom with their peers, they actively participate and the flipped learning approach could be applied to all types of situations such as standard or blend lectures.

The evolution of technology in education causes the emergence of new methodologies in language learning. In the digital age, new technologies are gaining traction, and technology resources are rapidly being integrated into the educational curricula (Nair and Yunus, 2021). These technology advancements ease the teacher’s role in preparing materials ahead of time and planning engaging and problem-solving activities in line with the twenty-first century skills. With the aid of technology, the flipped learning approach is an innovative and flexible teaching and learning pedagogy to be implemented during this pandemic. Although flipped learning can be used to teach the four skills of learning English (listening, speaking, reading, and writing), most english as a foreign language (EFL) students have difficulty speaking in front of the class or conversing with the teacher (Dasa et al., 2021). This approach promotes positive experiences in language acquisition, particularly in speaking skills. In this research, Blendspace is a tool used in this study to improve students’ speaking skills using the flipped learning approach.

### Blendspace in Improving Speaking Skills

**FIGURE 1:**
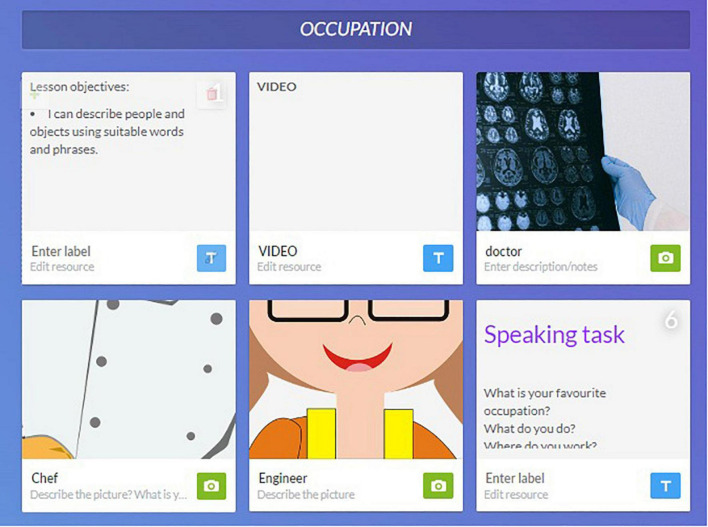
A complete Blendspace tool.

Blendspace is one of the interactive tools to organize and store materials in a single platform for the pupils. Blendspace comprises multimodal rich contents such as videos, presentations, textual links, and images to cater to different multiple intelligences and learning styles. In this research, the researcher utilized the Blendspace tool to improve speaking skills in the flipped learning approach. The flipped learning approach utilized technology to ensure pupils practice speaking in an interactive and fun manner. This promotes twenty-first century skills as pupils assess the lesson materials and complete the speaking tasks in Blendspace. We planned and created the lesson from the Blendspace website. The researcher used Blendspace as the medium to disseminate information and exercises during the “outside classroom” phase. This was done to arouse pupils’ interest in the flipped approach as they are guided by Blendspace learning styles. Pupils could easily assess Blendspace and regulate their own learning behavior. Differentiated lessons and built-in assessments were used to tackle speaking problems. An example of a complete Blendspace tool is shown in Figure 1.

The researcher planned and drafted the speaking contents according to their respective weeks. The lesson objectives were stated and some relevant videos were post. Each video lasted 3–7 min. The video was followed by a quiz to check their understanding and help to practice and reflect on their learning in line with Bloom’s Taxonomy. Pupils were be well-prepared and perform well in the speaking task. Each Blendspace has a speaking task as homework and the pupils can record their speaking task and send it to their teacher.

An innovative feature in Blendspace is multiple frames that could be viewed to teach a content and explore relationships between each content in a single platform. An interactive presentation could be used to present ideas and information to arouse pupils’ interest to participate and ease language skills in language learning. A study was conducted by Mansor and Yunus (2017) to gather participants’ responses on the use of Blendspace to improve pupils’ speaking skills. This survey increased the participants’ confidence level to speak well for their job interview. The findings obtained had provided positive impacts on the teaching and learning process of English language speaking skills. Blendspace creates an authentic learning experience for the participants. Therefore, we utilized the Blendspace tool to improve the speaking skills in the flipped learning approach.

There are a limited number of studies on the use of Blendspace tool. Findings proved that Blendspace is suitable in the flipped classroom as students are responsible for their own learning (Zainuddin and Perera, 2018; Zainuddin et al., 2019a,b). Hence, pupils gain autonomy and can learn at their own pace. Also, technology-integrated pedagogy provides boundless resources for education (Sun and Gao, 2019). As a result, to produce a better twenty-first century paradigm shift, teachers should use technology in the flipped learning strategy. Moreover, a study by Adnyani et al. (2018) was conducted by using Blendspace in a phonology class. On a total mark of 100, more than 70% of the pupils scored 76–100 marks and less than 30% of the pupils scored less than 76 marks by the use of the learning material, Blendspace. It was concluded that Blendspace is an effective tool in implementing phonology learning materials and improving their phonological knowledge. In addition, Blendspace was used in blended learning for the Public Sector Accounting course (Nor and Kasim, 2015). The purpose of this study was to determine how frequently accounting students used Blendspace. Blendspace’s improved usability helped educators to organize teaching and learning resources more easily.

Blendspace is suitable for all types of schooling levels. The flexibility of Blendspace enables teachers to design the right resources needed to equip their pupils with knowledge and skills to improve their speaking capabilities. In fact, Blendspace could be used to tackle other difficult subjects regardless of their school grade or university course. Numerous past studies on flipped learning have been published on undergraduate participants (Abdullah et al., 2019; Bezzazi, 2019; Joseph and Joy, 2019; Chen and Hwang, 2020; Yoon and Kim, 2020). Although many researchers have published and conducted their research on flipped learning, the majority of the studies were done on undergraduate participants, leaving a gap at the primary school level. Thus, it is crucial to know to what extent the flipped learning approach is suitable for the younger primary pupils and to tackle the speaking problems. The study is therefore designed to address the following research questions:

1. What is the significant difference in speaking achievement between before and after being taught using Blendspace?
2. What are the pupil’s perceptions of the flipped learning approach to improve pupils’ speaking skills?

## Materials and Methods

The research used a mixed-method whereby the aim of this research was to explore the benefits of flipped learning in improving speaking skills among the Year 3 pupils. The research participants were 40 Year 3 pupils in a primary school located in Klang. This research used a purposive sampling method in which the participants had weak proficiency levels after being assessed through the SBA. Pupils should also have access to technological gadgets so they could participate in this research. The research was conducted for over 7 weeks and pupils practiced their speaking skills with the aid of the Blendspace tool through the flipped learning approach.

The research procedures were divided into two sections, which are “outside class” and “inside class.” In the first section, “outside classroom,” the researcher created a WhatsApp group to post any important information and instruction to the research participants. In the beginning, all the participants were given a speaking pretest to assess their initial performance level. Then, the researcher posted the activity link of the LMS Blendspace in the WhatsApp group every week. There were five modules that we used throughout this research. Each module complements a topic in the Year 3 syllabus and was discussed each week subsequently. The researcher created contents for the Blendspace according to the respective modules.

The data were collected from three types of instruments, which are speaking pre–posttests, questionnaires, and interviews. The speaking test was focused to investigate whether there is any significant difference in speaking achievement before and after being taught using Blendspace. The pretest was given at the beginning of the study to assess the students’ ability and initial speaking performance level, while the posttest was given at the end to assess the students’ speaking performance level. In the pretest and posttest, pupils were asked to choose two speaking cards and then described the picture. The researcher recorded them according to the speaking rubric by Brown (2004). The rubric consists of five categories, which are grammar, vocabulary, comprehension, fluency, and pronunciation. The pupils were assessed and the scores were recorded.

Data were gathered through a questionnaire focusing on the pupil’s perceptions of the flipped learning approach to improve pupils’ speaking skills. The questionnaire was adapted from a survey by Johnson (2013). This questionnaire consists of 14 five-level Likert scale items. All the statements were oriented on a Likert scale ranging from 1 (strongly disagree), 2 (disagree), 3 (neutral), 4 (agree), and 5 (strongly agree). All items were presented in the English language followed by the Malay language. This was done to ensure that participants understood the questions and could make appropriate responses. The school’s Head of the Bahasa Melayu panel double-checked the back-to-back translation.

A semi-structured interview was used and follow-up questions were asked if needed. The interviews varied in length according to the understanding and the comfort level of the participants, which were the deciding factors of the interview process (Creswell, 2013). The interviews were conducted in two languages, English and Malay. This was done in consideration that pupils might not understand and answer the interview questions. We translated the interview protocol into Malay language and sought help from a teacher from the Malay Language department in the school.

Based on the data analysis, the speaking pretest and posttest were graded by a speaking rubric by Brown (2004). The rubric contains grammar, vocabulary, comprehension, fluency, and pronunciation. The results from the pretest and posttest were analyzed using a paired sample *t*-test. We utilized IBM SPSS version 26 software in this study. This was done to show the differences between the pretest and posttest. Based on the interrater reliability, the participants’ speaking performances were graded by two raters (the Head of the English Panel and a senior teacher experienced in the field of English language teaching). On the other hand, the analysis of the questionnaire was analyzed using descriptive statistics and the SPSS. The pupils’ responses were measured using means, standard deviations, and percentages to measure their perception of the flipped learning approach. Qualitative data analysis (interview method) was done based on the thematic analysis.

The validity and reliability of the study were piloted and the Cronbach alpha was 0.780, which was acceptable. This indicates that the questionnaire was highly reliable. The questionnaire was further double-checked and amended by two experienced panel experts in the subject of education. The experts were a lecturer from the English Department at IPG Bahasa Antarabangsa and an officer from Jabatan Pendidikan Negeri Sarawak. The validation of the questionnaire was done at the beginning of the research to ensure that the items could be understood and interpreted correctly by the research participants.

## Results

### Speaking Achievement Between Before and After Being Taught Using Blendspace

A paired sample *t*-test was conducted to analyze the results of the first research question. Table 1 shows the results of pretest and posttest.

**TABLE 1:** Mean scores of pre–posttests.

Test	Mean	Standard deviation
Speaking pretest	31.3250	7.99483
Speaking posttest	69.0000	12.04266

Based on the statistics, the findings indicate that the pupils showed improvement in their pretest and posttest. The results showed a significant increase in the marks of the pretest (*M* = 31.33, *SD* = 7.99) to post-test (*M* = 69.00, *SD* = 12.04), *t* (49) = −22.436, *p* < 0.05). This demonstrates that pupils’ speaking skills have improved after being taught with Blendspace. Table 2 shows the results of paired sample *t*-test.

**TABLE 2:** Paired sample test.

Paired differences					
	Mean	Standard deviation	t	df	Sig (2-tailed)
Speaking pretest/Speaking posttest	−37.67500	10.62022	−22.436	39	0.000

Assuming the level of significance (α) as 0.05 and the degree of freedom (*df*) as 39, the result showed a significant increase (*t* = −22.44, *p* = 0.000). The paired sample *t*-test results show that the participants scored higher scores on speaking posttests. The application of Blendspace in the flipped classroom had significantly impacted the pupils’ speaking skills. Meanwhile, the effect size was calculated using eta square. The effect size was 0.93 when representing a large effect size, indicating that Blendspace has a high effect in improving pupils’ speaking skills through the flipped learning approach.

### Pupil’s Perceptions of the Flipped Learning Approach to Improve Pupils’ Speaking Skills

The questionnaire and interview were administered to the participants to measure their perception of the flipped learning approach to improve their speaking skills. The pupils’ perception was divided into three constructs, which included participation, interaction, and enjoyment. It is important to note the differences and to understand to what extent the flipped learning approach could enhance their speaking skills. There are two distinct themes that emerged from the research data. The pupils were not exposed to the new flipped learning approach. They have been learning in the traditional classroom to practice their speaking skills. Furthermore, the pupils could only practice speaking inside the classroom where they are facilitated by a teacher.

Table 3 shows the overall mean of constructs that measure pupils’ perception about flipped learning approach to improve pupils’ speaking skills. The findings reported that participation has the highest mean (*M* = 4.045), followed by interaction (*M* = 3.931) and enjoyment (*M* = 3.880). This reflects the pupils’ perception about the flipped learning approach; hence, there is a need to identify to which extent this approach was effective to improve their speaking skills. Table 4 shows the frequencies and meaningful interpretations of the pupils’ participation aspect in the flipped learning approach.

**TABLE 3:** Mean of constructs.

Pupils’ perception	Mean	Interpretation
Participation	4.045	Agree
Interaction	3.931	Agree
Enjoyment	3.880	Agree

**TABLE 4:** Mean score and standard deviation of participation.

Item	Statement	*SD* (%)	D (%)	*N* (%)	A (%)	SA (%)	Mean	Interpretation
1	Flipped learning is a new strategy for me	0	2 5	4 10	16 40	18 45	4.2500	Strongly agree
2	Flipped learning increases my participation level during speaking practice	0	2 5	6 15	19 47.5	13 32.5	4.0750	Agree
3	I am more prepared to learn with the flipped learning approach	0	3 7.5	10 25	14 35	13 32.5	3.9250	Agree
8	I have the freedom to view the materials	0	1 2.5	9 22.5	18 45	12 30	4.0250	Agree
14	I prefer this new approach than the traditional face-to-face approach	0	1 2.5	12 30	15 37.5	12 30	3.9500	Agree
	Total						4.045	Agree

Based on Table 4, the pupils who discovered flipped learning as a new strategy (*M* = 4.250) participated often in speaking tasks (*M* = 4.075), were more prepared (*M* = 3.925), had the freedom to access materials (*M* = 4.025), and preferred the flipped approach over the traditional approach (*M* = 3.950). Based on the responses, the data illustrate that Item 1 elicited the strongest response from pupils, stating that “Flipped learning approach is a new strategy for me,” which has the highest average mean of *M* = 4.25. Eighteen participants (45%) strongly agreed and 16 participants (40%) agreed that flipped learning is a new strategy. There were four participants (10%) who responded neutral and only two participants (5%) disagreed with this statement. The majority of the participants discovered that the flipped learning approach is somewhat interesting and new to them, especially when it was being practiced during the COVID-19 pandemic.

Based on Table 5, the pupils agreed that interaction was important as it increases their interest to speak (*M* = 4.050), motivates them to practice speaking at their own pace (*M* = 3.87), increases their interest to interact with peers (*M* = 3.925), and increases their confidence level to speak (*M* = 3.87). The findings of all items indicated that the pupils had an average mean of291 (*M* = 3.931, *SD* = 0.836).

**TABLE 5:** Mean score and standard deviation of the interaction.

Item	Statement	*SD* (%)	D (%)	*N* (%)	A (%)	SA (%)	Mean	Interpretation
4	Flipped learning increases my interest to speak in English	0	3 7.5	4 10	21 52.5	12 30	4.0500	Agree
6	I can practice speaking skills at my own pace.	0	1 2.5	11 27.5	20 50	8 20	3.8750	Agree
7	I can interact more with my friends	0	1 2.5	14 35	12 30	13 32.5	3.9250	Agree
9	I am more confident to speak in English	0	0	17 42.5	11 27.5	12 30	3.8750	Agree
	Total						3.931	Agree

Item 4 elicited the highest mean value (*M* = 4.05) in the “Interaction” construct. Based on Item 4, “Flipped learning increases my interest to speak in English,” almost half of the participants (52.5%) agreed that they were interested to speak in English, followed by 30% of the participants who strongly agreed with the statement. The flipped learning approach provides a comfortable environment so that the participants could come out of their comfort zone of speaking in their mother tongue to speak in English. This showed a very positive attitude to practice speaking the English language. There was only a minimal response of disagreement with the statement, in which 10% of the participants were neutral and 7.5% disagreed. The overall interpretation indicates that the pupils agreed that they were excited and interested to speak through the flipped learning approach.

An interesting finding from this construct was found in Item 9 of the questionnaire. Based on Item 9, “I am more confident to speak in English,” there was no disagreement at all in the participants’ responses. Seventeen participants (42.5%) neither agreed nor disagreed with the statement. There were 12 participants (30%) who strongly agreed and 11 participants (27.5%) who agreed with this statement. The participants gained their confidence to speak in English as they were frequently exposed and monitored consistently throughout the flipped learning approach. As this research was conducted for 7 weeks, the participants were given the opportunity to interact with their peers through Google Meet sessions. They discussed and exchanged feedback based on the speaking topics. At first, the participants tried speaking chunks of words and combined their mother tongue (Bahasa Melayu) to answer questions and give feedback. Eventually, their speaking behavior changed and was noticed from the third week onward. Therefore, constant practice increased the pupils’ motivation and confidence level to improve their speaking skills. This shows that the majority of pupils agreed that the flipped learning approach increases their interaction as an improvement to practice speaking.

Based on Table 6, the overall mean and standard deviation (*M* = 3.88, *SD* = 0.904) indicated that pupils agreed and perceived the flipped learning approach as enjoyment to improve their speaking skills. The pupils enjoyed watching English videos (*M* = 3.575), Blendspace activities (*M* = 3.900), and the Blendspace tool (*M* = 3.900). Item 12, “Blendspace tool is easy to use,” recorded the highest mean score (*M* = 4.125). There were 14 participants (35%) who strongly agreed and 19 participants (47.5%) who agreed that they could use Blendspace easily. This could be due to the Blendspace layout, which was colorful and user-friendly. The participants liked the application of the Blendspace tool as it is more digitalized and attractive to use. The Blendspace tool aids the flipped learning approach to improve pupils’ speaking skills. However, there were six participants (15%) who neither agreed nor disagreed with the statement and there was one participant who strongly disagreed that Blendspace was easy to use. This could be due to the technical difficulties that they faced to login into Blendspace. Internet connection contributes a major role for the participants to enter Blendspace and assess the speaking materials easily.

**TABLE 6:** Mean score and standard deviation of enjoyment.

Item	Statement	*SD* (%)	D (%)	*N* (%)	A (%)	SA (%)	Mean	Interpretation
5	I like watching English videos.	0	8 20	10 25	13 32.5	9 22.5	3.5750	Agree
10	I enjoyed doing the activities on the Blendspace platform	1 2.5	2 5	7 17.5	20 50	10 25	3.9000	Agree
11	I like to use the Blendspace tool	0	2 5	12 30	14 35	12 30	3.9000	Agree
12	Blendspace tool is easy to use	1 2.5	0	6 15	19 47.5	14 35	4.1250	Agree
13	I would recommend flipped learning to my friends.	0	0	14 35	16 40	10 25	3.9000	Agree
	Total						3.88	Agree

Based on the interview data, six pupils mentioned their experience with Blendspace and the positive impacts they gained. As Blendspace contains many activities such as interactive videos, images, posters, quizzes, and speaking tasks, the pupils enjoyed completing the activities. Blendspace is very organized and the pupils learned actively in a fun learning environment. Similarly, they responded that they had fun learning with their peers although they were at home. This shows that Blendspace ensures pupils learn actively through the flipped learning approach. The role of Blendspace was to aid the flipped learning approach and to extend the teaching and learning process beyond the classroom for better engagement. Based on the interview responses, the pupils also liked Blendspace for multiple responses. The role of Blendspace was to aid the flipped learning approach and to extend the teaching and learning process beyond the classroom for better engagement. The responses are summarized in Table 7.

**TABLE 7:** Pupils’ preferences on Blendspace.

Participant	Responses
Santhana	It was very colorful and attractive very organized
Catherine	Very organized and can access all the lessons easily. I like the quiz and speaking tasks.
Samantha	I like to learn with Blendspace as we can log in into our own account. I am proud because I can use my name. I can assess the materials at anytime
Calvin	I like the variety of activities; videos, images, and homework. It is attractive and fun to learn
Joshua	Very colorful and I can watch the videos many times
John	I can comment on the post and see my friends’ comments too I like to play the quiz and enjoyed watching videos.
Christopher	can watch the video many times at my own pace. I don’t feel bored and like doing all the modules.
Gina	I can study the materials anytime and the layout of Blendspace is cool and fun to learn

Based on the responses, the majority of the pupils liked Blendspace due to its layout and functions. Since Blendspace was introduced for the first time, the pupils were excited and confused about how to use Blendspace through this flipped learning approach. It was a new experience to improve their speaking skills through Blendspace with the flipped learning approach. Hence, pupils need to be aware and upgrade themselves with digital skills and knowledge. This is crucial to increase their capability of using technological devices in the educational context.

On the other hand, active learning encourages positive impacts on pupils’ behavior in learning and gaining knowledge. Active learning improves pupils’ learning outcomes as well as their motivation and attitudes (Isaias et al., 2017). This complements the second pillar of the flipped learning approach, which is “Learning Culture” by the Flipped Learning Network (2014), which represents a student-centered approach. Pupils are actively constructing information and getting meaningful learning. In contrast to the traditional teacher-centered approach, in which the teacher is the major source of knowledge, this model is student-centered (Rahman et al., 2020).

## Discussion

As stated by Sudarmaji et al. (2021), the flipped classroom increased pupils’ speaking performance level in which they could preview the pre-class materials and then practice their speaking skills. Subsequently, this result supported the findings of Mansor and Yunus (2017) who reported that pupils had more opportunities to practice the language with the aid of Blendspace. They were more prepared in the speaking lesson with the aid of Blendspace. The Blendspace tool enhanced pupils’ active learning through the use of instructional videos, interactive quizzes, and speaking exercises. Pupils are free to learn at their own speed. This speaking improvement is in line with similar findings (Abdullah et al., 2019; AlKhoudary and AlKhoudary, 2019) that showed a significant difference between pretest and posttest by a paired sample *t*-test. The key to pupils’ success to improve their speaking skills was consistent speaking practice both inside and outside of the classroom. As a result, the flipped learning approach facilitates the use of Blendspace because students are responsible to practice the language, thus improving their speaking skills.

Moreover, the pupils were familiar with the context of the speaking tests, so they had many previous experiences and were exposed to many vocabularies in relation to the topics. The Blendspace tool increased pupils’ interest and motivation to complete the speaking tasks, which helped them to understand the speaking lesson meaningfully. A similar pattern of motivation results was obtained by Joseph and Joy (2019); Lie and Yunus (2019), Andujar et al. (2020), and Parra-González et al. (2020). To engage pupils in the teaching and learning activities beyond the classroom environment, digital tools and classrooms are required, which ensures pupils have access to online sources and platforms as well as to interact with their peers and teachers (Erdemir and Yangın-Ekşi, 2019). The results also indicated that pupils who used Blendspace at their own pace have increased their ability to communicate and comprehend the meaning.

The overall findings of this investigation measure pupils’ perception of the flipped learning approach to improve their speaking skills. The pupils’ perception was divided into three constructs, participation, interaction, and enjoyment. The findings reported that the highest mean obtained was found in the participation construct (*M* = 4.045), followed by interaction (*M* = 3.931) and enjoyment (*M* = 3.880). Pupils participate actively inside the classroom and outside the classroom to improve themselves. The interview is added to triangulate the data obtained. The interviews with the research participants showed that active learning and increased interaction inside and outside of the classroom facilitate the pupils’ involvement in this research. This increases pupils’ interest and preference for this flipped approach over the traditional approach. In this study, Blendspace acts as a tool to aid the flipped learning approach in increasing the participation level outside the classroom. This supports research findings from Zainuddin and Perera (2018) that a new technique is desperately needed to increase pupils’ participation in class in the flipped classroom.

Moreover, the pupils enjoyed watching videos on Blendspace and completing the speaking task. The pupils recorded their speaking tasks and sent the audio through WhatsApp. This inculcates fun learning to improve their speaking skill. This ensures pupils to self-regulate their own learning behavior to achieve the desired goal. In line with the previous findings on Blendspace by Zainuddin et al. (2019a,c) proved that Blendspace is suitable to improve one’s speaking skills in becoming self-directed learners. Pupils only speak English in the English language class, and because the majority of the students are Malay, they prefer to interact in Malay both inside and outside the classroom. This result ties well with a previous study by Nair et al. (2021), whereby the pupils only speak English in the English language class, and because the majority of the students are Malay, they prefer to interact in Malay both within and without the classroom.

In addition, the research findings highlighted that pupils showed positive responses toward the flipped learning approach. Similar findings were reported by Andujar et al. (2020), in which pupils showed good attitude and perception in the flipped learning approach. The findings of the study revealed that students responded positively to the flipped learning method, which was a novel strategy for the majority of the students. It was also reassuring to note that pupils were able to interact more with their peers despite the school closure. The flipped classroom allows students to interact both inside and outside of the classroom *via* Google Meet and Blendspace. The results were found to be consistent with that of Abedi et al. (2019), who viewed pupils to be more motivated and independent with the flipped learning approach. Hence, this ensures that pupils should be more engaged and be prepared before class (Bezzazi, 2019).

Undeniably, the flipped learning approach helps both teachers and pupils to store and assess materials easily and learn at their own pace. The pupils are responsible for their own success in learning through the Blendspace tool. This ensures pupils maximize their self-regulating skills and interaction with their peers both inside and outside the classroom. Despite the pandemic, pupils are could to interact with one another to complete the speaking tasks. Hence, this research yields positive findings in improving one’s speaking skills.

The findings for the first research question revealed that the pupils showed improvement in their speaking achievement with the usage of Blendspace. The pupils were motivated to speak the language. It is clear that the mean scores of posttest results showed that the utilization of Blendspace proved to greatly improve speaking skills among the Year 3 pupils. Blendspace does not only act as a platform that stores speaking materials but also contains various types of quizzes and speaking tasks. This could arouse pupils’ interest to practice speaking at home. Hence, the application of Blendspace could increase pupils’ speaking skills.

The findings for the second research question revealed that pupils had a positive perception about the flipped learning approach to improve pupils’ speaking skills. The pupils’ perception was divided into three constructs, participation, interaction, and enjoyment. The findings reported that the participation construct has the highest mean (*M* = 4.045), followed by interaction (*M* = 3.931) and enjoyment (*M* = 3.880). Therefore, the flipped learning approach does not only improve pupils’ speaking skills but also ensure active participation to speak confidently.

## Conclusion

The findings of this research consist of important implications that could contribute to the field of learners’ language acquisition in the context of the flipped learning approach. The flipped learning approach utilizes Blendspace as a tool to improve pupils’ speaking skills. This research increased pupils’ motivation to learn as they are immersed with digital technology in this twenty-first century education. Gamification could be used to enhance pupils’ motivation and engagement to learn better. This increases pupils’ productivity level and problem-solving skills. Interactive game features and rewards such as badges and points contribute to their active participation and reflective thinking. The flipped learning approach could utilize gamification as one of the components to improve pupils’ speaking skills. The flipped learning approach is a flexible teaching and learning method that should be implemented by educators for better engagement. Thus, this study serves as a positive paradigm shift in sustaining the teaching and learning processes during the COVID-19 pandemic or any other worldwide-related situations. Similarly, this approach enables pupils to have quality time within as well as outside the classroom to practice their speaking skills. The teaching and learning of English could be more motivating, and more language activities could be implemented without worrying about time-constraint factors. This is undeniably important as consistent speaking practice would lower pupils’ speaking anxiety and increase their confidence level.

The second major impact of how this explicit focus can be manifested is through active and flipped instruction. The key is for teachers to include the HOTS aspect during classroom interaction and promote open discussions. Classroom interactions increased pupils’ confidence level and motivate them to practice speaking with the guidance of the teacher. As the study also involved inside and outside classroom interactions, pupils were able to learn at their own pace. This gives a big impact as it shapes pupils’ attitudes to be more mature and responsible in their learning process. Pupils were able to embrace the learning process. This study has proved favorable results in pupils’ active participation and classroom interaction to improve their speaking skills.

There are two limitations to this study, the scope of the research and the sample size. This study was limited to English speaking skills in Dokumen Standard Kurikulum dan Pentaksiran (DSKP) Year 3 syllabus. The content of the lesson was based on the information communicating simple information intelligibly, the use of appropriate communication strategies, and describing people and things clearly as stated in the Year 3 Dokumen Standard Kurikulum dan Pentaksiran (Sekolah Kebangsaan) English curriculum specifications. Thus, different findings and lesson content must be modified to suit other year pupils in improving their speaking skills. Second, the sample size was small, which was only 40 pupils. A large-scale replication needs to be done to obtain better precision and for generalization purpose.

The flipped learning technique yields promising results in terms of improving students’ speaking abilities. However, there are a few suggestions that future researchers could use to broaden the area of their research. First, teachers need to be given proper training on ways to implement the flipped learning approach in their English lessons. This is important as the approach could maximize the opportunities to coach pupils to achieve the learning objectives. The government and teacher training institutions should conduct workshops and trainings to educate teachers on the implementation and strategies of the flipped learning approach. Hence, teachers could monitor each pupil and do amendments to suit pupils’ learning styles in line with twenty-first century education.

Second, more research should be done on primary school pupils. A majority of the studies in regard to the flipped learning approach were conducted among undergraduate participants. Thus, research on primary school pupils should be strengthened so that pupils could be nurtured from a young age to learn and master language skills easily, particularly speaking skills. This could also be a considerable potential to tackle pupils’ problems in other subjects as well.

Third, this current study focuses on one language skill, which was to improve pupils’ speaking skills. Future studies should be done to tackle problems in other language skills such as listening skills, reading skills, and writing skills. Hence, pupils could improve other language skills holistically. This research allows pupils to increase their speaking skills and enjoy the teaching and learning process with a new flexible approach. We hope that the findings of the study will be useful for teachers and policymakers to guide pupils in improving their speaking skills.

## Data Availability Statement

The original contributions presented in this study are included in the article/supplementary material, further inquiries can be directed to the corresponding author/s.

## Ethics Statement

The studies involving human participants were reviewed and approved by the Universiti Kebangsaan Malaysia. Written informed consent to participate in this study was provided by the participants’ legal guardian/next of kin.

## Author Contributions

CS: conceptualization, design, analysis, and writing. MY: editing and reviewing, supervision, grant management, and final approval. Both authors contributed to the article and approved the submitted version.

## Conflict of Interest

The authors declare that the research was conducted in the absence of any commercial or financial relationships that could be construed as a potential conflict of interest.

## Publisher’s Note

All claims expressed in this article are solely those of the authors and do not necessarily represent those of their affiliated organizations, or those of the publisher, the editors and the reviewers. Any product that may be evaluated in this article, or claim that may be made by its manufacturer, is not guaranteed or endorsed by the publisher.
